# Energy-Optimal Latency-Constrained Application Offloading in Mobile-Edge Computing

**DOI:** 10.3390/s20113064

**Published:** 2020-05-28

**Authors:** Xiaohui Gu, Chen Ji, Guoan Zhang

**Affiliations:** School of information science and technology, Nantong University, Nantong 226019, China; 17110013@yjs.ntu.edu.cn (X.G.); gzhang@ntu.edu.cn (G.Z.)

**Keywords:** mobile-edge computing, mobile application offloading, partial offloading, channel condition, energy-latency trade-off

## Abstract

Mobile-edge computation offloading (MECO) is a promising emerging technology for battery savings in mobile devices (MD) and/or in latency reduction in the execution of applications by (either total or partial) offloading highly demanding applications from MDs to nearby servers such as base stations. In this paper, we provide an offloading strategy for the joint optimization of the communication and computational resources by considering the blue trade-off between energy consumption and latency. The strategy is formulated as the solution to an optimization problem that minimizes the total energy consumption while satisfying the execution delay limit (or deadline). In the solution, the optimal transmission power and rate and the optimal fraction of the task to be offloaded are analytically derived to meet the optimization objective. We further establish the conditions under which the binary decisions (full-offloading and no offloading) are optimal. We also explore how such system parameters as the latency constraint, task complexity, and local computing power affect the offloading strategy. Finally, the simulation results demonstrate the behavior of the proposed strategy and verify its energy efficiency.

## 1. Introduction

For the past several years, mobile devices (MDs), such as smartphones, handheld game consoles, and vehicle multi-media computers, have become virtually ubiquitous and an increasing number of new mobile applications, such as augmented reality (AR), image processing, natural language processing, face recognition, and interactive gaming, have emerged and been the focus of considerable attention [1,2]. These mobile applications are typically latency-sensitive, computation-intensive, and have high energy consumption characteristics. However, under the constraint of physical size, MDs have limited resources, which restricts their battery life and computational capacities [3,4]. Recent studies have shown that mobile-edge computation offloading (MECO) technology provides a promising opportunity for effectively overcoming the hardware limitations and energy consumption problems of MDs, by offloading computing-intensive tasks to adjacent clouds at the edges of mobile networks.

In particular, mobile-edge computing (MEC) offers cloud computing capabilities at the very edge of the mobile networks by deploying MEC servers with sufficient computational resources at base stations (BSs), which thus improves the computational efficiency and reduces the latency, and it has drawn significant attention from both academia and industry [5]. The paradigm shifts from cloud computing to MEC can effectively reduce the backhaul latency and energy consumption, as well as support a more flexible infrastructure in a more cost-effective way. For example, the work in [6] used unmanned aerial vehicles (UAVs) to help device-to-device (D2D) wireless networks [7]. Furthermore, MEC together with virtual machine (VM) migration can effectively increase the scalability while reducing service delay [8,9]. Owing to these advantages, MEC has attracted extensive research attention from various aspects.

Because computational performance and energy consumption are competing for resources and are critical for mobile users [10], effective computation offloading schemes have become prominent for MEC systems. Basically, a decision on computation offloading may result in binary offloading and partial offloading, which are closely related to the application model/type. It determines whether full or partial offloading is applicable, what could be offloaded, and how [3]. A highly integrated or relatively simple task cannot be partitioned and has to be executed as a whole either locally at the mobile device or offloaded to the MEC server, called binary offloading. In practice, many mobile applications are composed of multiple procedures/components, making it possible to partition the program into two parts with one executed at the mobile device and the other offloaded for edge execution, called partial offloading. Although the former is easier to implement, for a very large dataset, by offloading critical subtasks to the MEC servers, partial offloading can help to reduce the latency and energy consumption on the local devices more flexibly and effectively [3]. However, partial offloading is a very complex process affected by different factors, i.e., whether the applications can be divided into offloadable parts or not; the offloadable part may differ in the amount of data and required computation; how to decide which parts could be offloaded to the MEC; the components of applications that need input from some others; and parallel offloading may not be applicable. Fortunately, these factors have been discussed and studied in many works [11,12,13,14,15,16,17,18,19].

For the offloading strategy, Bi et al. [20] studied the binary scheme in the wireless powered MEC network that consisted of one server and several users, where the binary policy was adopted for maximizing the weighted sum computational rate. Zhang et al., in [21], used the auction theory to model the matching relationship between the MEC server and the MDs, so as to offload tasks to the optimal MEC server. Al-Shuwaili et al., in [22], jointly allocated communication and computational resources to minimize the total MD energy consumption under latency constraints by successive convex approximation. In [23], by considering the data arrival time instants and computational deadlines, You et al. proposed an energy-efficient resource management policy for MECO systems and formulated an optimization strategy that minimized the total mobile energy consumption. For partial offloading, the work in [24] investigated MEC systems with one energy harvesting (EH) device and proposed an effective dynamic computation offloading algorithm to minimize the execution cost. In [25], You et al. studied the energy saving partial computation offloading problem, for a multiuser MECO system based on time-division multiple access (TDMA) and orthogonal frequency-division multiple access (OFDMA). In [26], Wang et al. also considered the energy consumption minimization problem in a scenario with one MEC server and multiple users. They also employed wireless power transfer to further alleviate the burden on battery usage. Furthermore, the work in [27] implemented a cooperative communication system that had three nodes, in which one of them acted as the helper for relaying and computing. Lastly, the work in [28] considered both the fronthaul and backhaul link transmission and offloading in a small-cell architecture.

However, some of above literature only focused on the performance indicator of latency, while this paper aims at minimizing the energy consumption, which is critical for power-limited devices, especially for a device with lower battery energy. Furthermore, in the problem formulation, the previously mentioned works [25,26] only considered the local computing time and transmission time, but the server computing time was neglected; thus, they fell short of accuracy for scenarios in which the MDs needed to offload computation-intensive jobs to MEC servers with limited computing capacity. Moreover, most of the works performed so far performed the management aspects, the experimental evaluation of energy savings associated with offloading, and/or the definition of an offloading criterion that considered the energy cost of the radio interface (e.g., LTE or WiFi), but without optimizing the energy cost of the data transfer according to the current channel conditions. Notice, however, that depending not only on the application, but also on the current channel conditions, the best strategy concerning the offloading process may be different. Considering minimizing the energy consumption at the MD for delay-constrained offloading jobs, an optimization problem can be formulated to allocate the computation and communication resources jointly in the described MECO system.

In this paper, we pursue an energy-efficient MEC offloading design considering the partial and binary strategies, aiming to minimize the energy consumption of the mobile application while ensuring that the computational task can be successfully finished with a predefined hard limit of execution time. We derive the analytical solution for the single-MD to the MEC case, which can be generalized to the multi-MD case in later research. Our work presents some main differences from previous works. First, we introduce an offloading model that includes energy consumption at MD and processing time at the server side, while these offloading parameters were neglected in previous works such as [24,27]. Second, instead of considering that the application is run either totally in the cloud or totally in the MD, we include an optimization variable as the quantity of data to be processed on each side to allow a partial trade-off between local computing and offloading. Finally, in contrast with previous works, our approach allows for adapting the transmission strategy to the current channel state as perceived by the MD in the uplink.

The main technical contributions of this paper are as follows.

1. Based on the offloading model above, we formulate an offloading-optimization problem that minimizes the MD energy usage while ensuring the task is completed within a prescribed deadline, by jointly optimizing the transmitting time and offloading ratio.
2. We transform the latency-constrained problem into a two-stage optimization problem, which can be analytically solved using standard convex optimization techniques. In the solution, a channel condition threshold is derived above which full offloading is the optimal decision, whereas below the threshold, partial offloading is performed to trade off between the time and energy cost of offloading. For the partial offloading policy, the optimal transmission time and offloading ratio are derived in closed form expression.
3. This paper also discusses in detail various practical aspects of the offloading strategy, including the conditions under which total offloading or non offloading is optimal, the minimum admissible latency constraint that renders the problem feasible, and how the system parameters affect the offloading decision, including the energy efficiency of MD, the task complexity, and the computing capacity of local devices.

The remainder of this paper is organized as follows. In Section 2, the communication and computational model in mobile execution and MEC execution are presented, respectively. In Section 3, the optimization problem is formulated and solved for optimal partial offloading, while binary offloading decisions are also discussed under different channel conditions. The effects on the offloading strategy of a number of system parameters are analyzed in detail in Section 4. Finally, some simulation results and conclusions are provided in Section 5 and Section 6, respectively.

## 2. System Model

**Figure 1:**
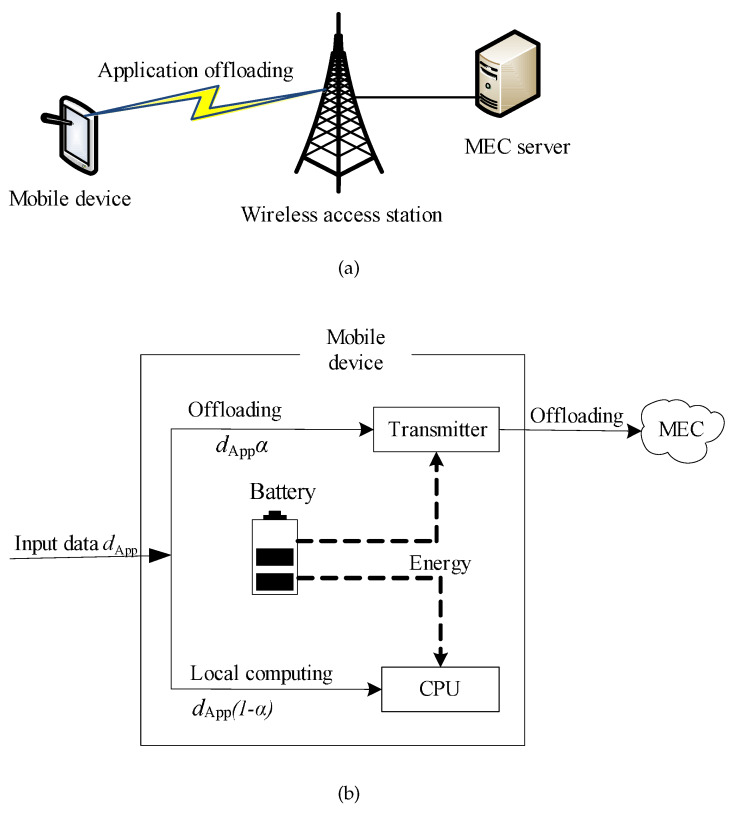
System model. (**a**) Mobile-edge computing (MEC) platform. (**b**) The offloading workflow.

As shown in Figure 1a, the MEC server is a computing device installed at a wireless access station. The MD is provided with wireless access to the computational resources located in proximal servers. By assigning the computing-intensive applications to the BS, it can help mobile users improve the computing performance. The pioneering literature has extensively studied mobile networking and mobile-edge computing models (e.g., [23,29,30,31]), which provide useful insights and make performance analysis tractable. Based on these results, we adopted a canonical model that captured the essentials of a typical mobile application. Although it is possible to build a system model to depict the various aspects of MEC in detail, such a model could be too complex to be treated analytically and is difficult for practical systems. Specifically, in this paper, the mobile application is abstracted into a profile with three parameters [22,23], including:

- Input data size $d_{\mathrm{App}}$: the number of data bits as the input to the application;
- Required CPU cycles $c_{\mathrm{App}}$: the number of CPU cycles required to complete the application;
- Application completion deadline $T_{\mathrm{Max}}$: the maximum latency, before which the application should be completed.

For the partial offloading policy, let α represent the ratio of the offloaded data to the full data size, and the offloaded data size of the mobile application can be denoted as $l_{\mathrm{Off}}=d_{\mathrm{App}}\alpha$. We consider the application scenario to be quasi-static, where the environment at MD will not change during the computation offloading period.

Notice that the input data size $d_{\mathrm{App}}$, the required CPU cycles $c_{\mathrm{App}}$, and the application completion deadline $T_{\mathrm{Max}}$ may have an impact on the energy consumption of mobile applications. With more data input, more CPU cycles, and/or a more stringent completion deadline, the energy consumption will be higher. Therefore, it will be beneficial to offload computation-intensive tasks to the MEC servers.

### 2.1. Communication Model

We first introduce the communication model between the MD and the MEC platform. The computation offloading policy was carried out based on energy consumption and completion time. The transmission power for the MD is denoted as $p_{\mathrm{Tr}}$, and $g_{s}$ represents the channel gain of the BS. The uplink data rate $r_{\mathrm{Tr}}$ for computation offloading of the MD is given by:

(1) $$r_{\mathrm{Tr}}=B\log_{2}\left(1+\frac{p_{\mathrm{Tr}}g_{s}^{2}}{(N_{0}+I)B}\right),$$

where $N_{0}$ and *I* represent the power spectrum density of additive white Gaussian noise and the interference signal, respectively. Letting *B* be the bandwidth of the channel, at the MEC, the received signal power can be denoted by a function of data rate *r*:

(2) $$h\left(r\right)=\left(N_{0}+I\right)B\left(2^{\frac{r}{B}}-1\right),$$

which is monotonically increasing and convex for r>0. The offloading transmission rate can be denoted as:

(3) $$r_{\mathrm{Tr}}=\frac{d_{\mathrm{App}}}{t_{\mathrm{Tr}}},$$

where $t_{\mathrm{Tr}}$ is the transmission time of the MD for offloading the input data size $d_{\mathrm{App}}$. Then, the transmission power $p_{\mathrm{Tr}}$ can be calculated by combining (2) and (3):

(4) $$p_{\mathrm{Tr}}=\frac{1}{g_{s}^{2}}h\left(\frac{d_{\mathrm{App}}}{t_{\mathrm{Tr}}}\right).$$

### 2.2. Computation Model

(1) Local computing: With the local computing approach, the MD executes the application locally on the MD. Let $h_{\mathrm{MD}}$ denote the computational capability (i.e., CPU cycles per second) of the MD, and accordingly, the completion time for local computing is defined as:

(5) $$t_{\mathrm{Lo}}=\frac{c_{\mathrm{App}}}{h_{\mathrm{MD}}},$$

For the local computation energy, we have:

(6) $$e_{\mathrm{Lo}}=f_{\mathrm{MD}}\;c_{\mathrm{App}},$$

where $f_{\mathrm{MD}}$ is the consumed energy per CPU cycle for the MD. Besides, $f_{\mathrm{MD}}$ indicates the energy efficiency of the MD.

(2) Edge computing: With the edge computing approach, the MD is allowed to offload its computational load to a nearby MEC server. Subsequently, the server performs the offloaded task and feeds the result back to the MD. Therefore, in the overall offloading, the following three consecutive phases are included: (i) transmitting phase, (ii) computing phase, and (iii) receiving phase. According to the communication model, the transmission time and energy consumption of the MD sending its computational load to the MEC server are calculated by:

(7) $$t_{\mathrm{Tr}}=\frac{d_{\mathrm{App}}}{r_{\mathrm{Tr}}},$$

and:

(8) $$e_{\mathrm{Tr}}=p_{\mathrm{Tr}}\;t_{\mathrm{Tr}}.$$

The execution time for the computing load $d_{\mathrm{App}}$ on the MEC server is computed by:

(9) $$t_{\mathrm{Ser}}=\frac{c_{\mathrm{App}}}{h_{\mathrm{MEC}}},$$

where $h_{\mathrm{MEC}}$ denotes the computational capacity of the MEC server.

For the edge computing approach, the completion time and energy consumption of the mobile application are formulated as:

(10) $$t_{\mathrm{Off}}=t_{\mathrm{Tr}}+t_{\mathrm{Ser}}+t_{\mathrm{Rec}},$$

and:

(11) $$e_{\mathrm{Off}}=e_{\mathrm{Tr}}+e_{\mathrm{Rec}},$$

where $t_{\mathrm{Rec}}$ and $e_{\mathrm{Rec}}$ denote, respectively, the time and energy required at the MD side when receiving the computational outcome from the MEC server. As in the existing works [32,33], the receiving time $t_{\mathrm{Rec}}$ and the receiving energy $e_{\mathrm{Rec}}$ can be ignored because for many applications, such as face recognition and car barrier detection, the data size of the result is relatively much smaller than the size of the input. In this paper, we consider the energy consumption on the MD side, while our future work will consider the execution energy consumption of the MEC server.

Generally, we can assume that the server has higher computational capacity than the mobile device, i.e., $h_{\mathrm{MEC}}>h_{\mathrm{MD}}$. Therefore, if a job is fully offloaded to the MEC server, the time saved in the computation phase can be expressed as:

(12) $$T_{\mathrm{Sav}}=c_{\mathrm{App}}\left(\frac{1}{h_{\mathrm{MD}}}-\frac{1}{h_{\mathrm{MEC}}}\right).$$

### 2.3. Partial Offloading Model

Next, we introduce the partial offloading model. As in [22,33], we apply a linear model in this paper and the MD enables programs to process computing tasks sequentially. Here, α is the offloading ratio, which is the proportion of the task data offloaded to the MEC server, as shown in Figure 1b. The offloading data rate is $r_{\mathrm{Tr}}=\frac{d_{\mathrm{App}}}{t_{\mathrm{Tr}}}$, the offloading transmit power $p_{\mathrm{Tr}}$, and the partial offloading time $t_{\mathrm{Off}}\alpha$. Therefore, the total energy consumption of the MD includes both local computing consumption and partial offloading consumption, which is expressed as:

(13) $$\begin{array}{cc} e_{\mathrm{Tot}} & =e_{\mathrm{Off}}\alpha +e_{\mathrm{Lo}}\left(1-\alpha \right), \end{array}$$

(14) $$\begin{array}{cc} \; & =p_{\mathrm{Tr}}t_{\mathrm{Tr}}\alpha +f_{\mathrm{MD}}c_{\mathrm{App}}\left(1-\alpha \right), \end{array}$$

Because we consider the computational performance as well, the completion time of the MD can be denoted as:

(15) $$t_{\mathrm{Tot}}=t_{\mathrm{Off}}\alpha +t_{\mathrm{Lo}}\left(1-\alpha \right).$$

The completion time above also includes the partial offloading time and the local computing time, which are proportional to the original full offloading time and local-computing time, respectively.

By the definitions of $e_{\mathrm{Tot}}$ and $t_{\mathrm{Tot}}$, the overall energy consumption of the MD is $e_{\mathrm{Tot}}$, for the task completed duration $t_{\mathrm{Tot}}$.

## 3. Joint Optimization of the Communication and Computation Resources with Partial Offloading

Based on the offloading model established in the previous section, we are now ready to optimize an offloading policy. Our goal was to find a policy that satisfies the hard delay constraints with minimal energy consumption. The steps were carried out by analytical means, and solutions were formulated under different channel conditions and computing profiles.

### 3.1. Optimization Formulation

Now, we present the optimization problem of minimizing the energy consumption at the MD while satisfying the hard delay constraint. As a first step, the total energy is a monotonically decreasing function of transmit time, as shown in Lemma 1:

**Lemma** **1.** *For any fixed* 0≤α≤1 *, the total energy* $e_{\mathrm{Tot}}$ *is monotonically decreasing with* $t_{\mathrm{Tr}}$ *.*

**Proof.**  Please see Appendix A. □

Lemma 1 proves that energy can be saved by extending the full offloading time under a fixed offloading ratio, till reaching the time constraint.

The optimization objective is to find the offloading policy to minimize the total energy consumption for the MD. It can be noted that $e_{\mathrm{Tot}}$ in (13) and $t_{\mathrm{Tot}}$ in (15) are affine functions of α. To simplify the problem, we let α and $t_{\mathrm{Tr}}$ be the optimization variables, and the optimal $p_{\mathrm{Tr}}^{*}$ can be calculated from $t_{\mathrm{Tr}}^{*}$ by Equation (4). Hence, the energy consumption minimization problem for the MD is formulated as:

(16) $$\begin{array}{cc} \; & \min_{\left\{t_{\mathrm{Tr}},\alpha \right\}}\;p_{\mathrm{Tr}}\;t_{\mathrm{Tr}}\;\alpha +f_{\mathrm{MD}}c_{\mathrm{App}}\left(1-\alpha \right)\\ s.t.\; & \left(t_{\mathrm{Tr}}+\frac{c_{\mathrm{App}}}{h_{\mathrm{MEC}}}\right)\alpha +\frac{c_{\mathrm{App}}}{h_{\mathrm{MD}}}\left(1-\alpha \right)\leq T_{\mathrm{Max}}. \end{array}$$

The constraint in (16) specifies that the total completion time of the application bits is bounded by the maximum affordable latency $T_{\mathrm{Max}}$.

It is shown from (16) that the primal optimization problem is affected by multiple parameters from both the communication and computational aspects. Of the parameters, the channel gain $g_{s}$ is crucial because it determines the quality of the wireless channel and, hence, the cost of application offloading in terms of energy.

### 3.2. Offloading Policy in Good Channel Conditions

In good channel conditions, a high-data-rate link can be established and maintained at a low energy cost. In addition, the MEC servers have greater computing capacity compared to the local MDs. Therefore, it is of interest to determine analytically when to perform full offloading to take advantage of high-performance servers and good channel conditions. We have the following assertion:

**Assertion** **1.** *There exists channel gain threshold* $g_{\mathrm{Th}}$ *that, for a channel gain better than threshold* $g_{\mathrm{Th}}$ *, full offloading with* $\alpha^{*}=1$ *is energy-optimal under any latency constraint, and:*

(17) $$g_{\mathrm{Th}}=\sqrt{\frac{\left(N_{0}+I\right)BT_{\mathrm{Sav}}\left(2^{\frac{d_{\mathrm{App}}}{BT_{\mathrm{Sav}}}}-1\right)}{f_{\mathrm{MD}}c_{\mathrm{App}}}}.$$

**Proof.**  Please see Appendix B. □

To satisfy the hard latency constraint at minimum energy cost, one can substitute $\alpha^{*}=1$ into (15) and apply Lemma 1. The optimal transmission time $t_{\mathrm{Tr}}^{*}$ for $g_{s}>g_{\mathrm{Th}}$ is given by:

(18) $$t_{\mathrm{Tr}}^{*}=T_{\mathrm{Max}}-\frac{c_{\mathrm{App}}}{h_{\mathrm{MEC}}},$$

and $p_{\mathrm{Tr}}^{*}$ can be computed using (4).

### 3.3. Offloading Policy for the Channel Condition below the Threshold

On the other hand, let us consider the case $g_{s}\leq g_{\mathrm{Th}}$. In that situation, it takes more energy to overcome the propagation loss to offload a task to the MEC server ($e_{\mathrm{Off}}\geq e_{\mathrm{Lo}}$). The objective function of (16) has the following features:

(19) $$\begin{array}{cc} e_{\mathrm{Tot}} & =e_{\mathrm{Off}}\alpha +e_{\mathrm{Lo}}\left(1-\alpha \right) \end{array}$$

(20) $$\begin{array}{cc} \; & =p_{\mathrm{Tr}}t_{\mathrm{Tr}}\alpha +f_{\mathrm{MD}}c_{\mathrm{App}}\left(1-\alpha \right)\\ \; & =\frac{\left(N_{0}+I\right)\;B\;t_{\mathrm{Tr}}}{g_{s}^{2}}\left(2^{\frac{d_{\mathrm{App}}}{Bt_{\mathrm{Tr}}}}-1\right)\alpha \end{array}$$

(21) $$\begin{array}{cc} \; & \;+f_{\mathrm{MD}}c_{\mathrm{App}}\left(1-\alpha \right), \end{array}$$

and:

(22) $$\frac{\partial^{2}e_{\mathrm{Tot}}}{\partial t_{\mathrm{Tr}}^{2}}=\frac{\alpha \left(N_{0}+I\right)2^{\frac{d_{\mathrm{App}}}{Bt_{\mathrm{Tr}}}}d_{\mathrm{App}}^{2}r_{\mathrm{Tr}}^{2}{\left(\ln2\right)}^{2}}{Bg_{s}^{2}t_{\mathrm{Tr}}^{3}}>0.$$

It can be observed that Problem (16) is non-convex and hence is challenging to solve directly.

**Assertion** **2.** *Problem (16) can be transformed into a two stage optimization problem, by iteratively finding the optimal* $\alpha^{*}$ *and* $t_{\mathrm{Tr}}^{*}$ *.*

**Proof.**  Since $\min_{\left\{t_{\mathrm{Tr}},\alpha \right\}}\;p_{\mathrm{Tr}}\;t_{\mathrm{Tr}}\;\alpha +f_{\mathrm{MD}}c_{\mathrm{App}}\left(1-\alpha \right)$ is equivalent to $\min_{t_{\mathrm{Tr}}}\min_{\alpha }\;p_{\mathrm{Tr}}\;t_{\mathrm{Tr}}\;\alpha +f_{\mathrm{MD}}c_{\mathrm{App}}\left(1-\alpha \right)$, one can first solve α for any specific value of $t_{\mathrm{Tr}}$ and, next, substitute the optimal $\alpha^{*}$ into (16) to find the optimal $t_{\mathrm{Tr}}^{*}$. Therefore, in the first sub-problem (23), we optimize the offloading ratio α for a given $t_{\mathrm{Tr}}$. In the second sub-problem (29), we aim to find the optimal $t_{\mathrm{Tr}}^{*}$, by substituting $\alpha^{*}$ into the original problem. □

The first sub-problem is given by:

(23) $$\begin{array}{cc} \; & \min_{\alpha }\;p_{\mathrm{Tr}}\;t_{\mathrm{Tr}}\;\alpha +f_{\mathrm{MD}}c_{\mathrm{App}}\left(1-\alpha \right)\\ s.t.\; & \left(t_{\mathrm{Tr}}+\frac{c_{\mathrm{App}}}{h_{\mathrm{MEC}}}\right)\alpha +\frac{c_{\mathrm{App}}}{h_{\mathrm{MD}}}\left(1-\alpha \right)\leq T_{\mathrm{Max}}. \end{array}$$

Therefore, we define function h(α):

(24) $$h\left(\alpha \right)≜e_{\mathrm{Tot}}.$$

It is obvious that h(α) is convex and non-decreasing monotonically with respect to α, since $e_{\mathrm{Off}}\geq e_{\mathrm{Lo}}$ under the condition where $g_{s}\leq g_{\mathrm{Th}}$. Furthermore, from the latency constraint, we can find $\alpha \geq \frac{t_{\mathrm{Lo}}-T_{\mathrm{Max}}}{T_{\mathrm{Sav}}-t_{\mathrm{Tr}}}$, under the default condition $t_{\mathrm{Lo}}\geq T_{\mathrm{Max}}$ and $T_{\mathrm{Sav}}\geq t_{\mathrm{Tr}}$ (When $g_{s}\leq g_{\mathrm{Th}}$, it costs more energy offloading data to remote servers than local computing. Therefore, if $t_{\mathrm{Lo}}<T_{\mathrm{Max}}$, the local computing is optimal; otherwise, partial offloading is energy-optimal.).

Hence, for a given $t_{\mathrm{Tr}}$, the optimal $\alpha^{*}$ is given by:

(25) $$\alpha^{*}=\frac{t_{\mathrm{Lo}}-T_{\mathrm{Max}}}{T_{\mathrm{Sav}}-t_{\mathrm{Tr}}}.$$

Next, we substitute (25) into the main problem and solve $t_{\mathrm{Tr}}$. We define:

(26) $$g\left(t_{\mathrm{Tr}}\right)≜\alpha^{*}=\frac{t_{\mathrm{Lo}}-T_{\mathrm{Max}}}{T_{\mathrm{Sav}}-t_{\mathrm{Tr}}},$$

and its second-order derivative is:

(27) $$\frac{\partial^{2}g\left(t_{\mathrm{Tr}}\right)}{\partial t_{\mathrm{Tr}}^{2}}=\frac{2(t_{\mathrm{Lo}}-T_{\mathrm{Max}})}{{\left(T_{\mathrm{Sav}}-t_{\mathrm{Tr}}\right)}^{3}}\geq 0$$

Therefore, $g(t_{\mathrm{Tr}})$ is convex with respect to $t_{\mathrm{Tr}}$. From all the above, the composite function $f\left(t_{\mathrm{Tr}}\right)=h\left(g\left(t_{\mathrm{Tr}}\right)\right)$ is convex [34] (chapter 3). By substituting (25) into (16),

(28) $$\begin{array}{cc} f(t_{\mathrm{Tr}})= & \frac{\left(T_{\mathrm{Max}}-t_{\mathrm{Lo}}\right)t_{\mathrm{Tr}}}{g_{s}^{2}\left(t_{\mathrm{Tr}}-T_{\mathrm{Sav}}\right)}h\left(\frac{d_{\mathrm{App}}}{t_{\mathrm{Tr}}}\right)+f_{\mathrm{MD}}c_{\mathrm{App}}\left(1-\frac{T_{\mathrm{Max}}-t\mathrm{Lo}}{t_{\mathrm{Tr}}-T_{\mathrm{Sav}}}\right). \end{array}$$

we can now solve the convex sub-problem that minimizes $f(t_{\mathrm{Tr}})$, thanks to the fact that $f(t_{\mathrm{Tr}})$ is convex. The optimal $t_{\mathrm{Tr}}^{*}$ is to be found by solving the second sub-problem:

(29) $$\min_{t_{\mathrm{Tr}}>0}\;f\left(t_{\mathrm{Tr}}\right)$$

#### Solution to the Sub-Problem

Next we solve the convex problem (29) using standard convex optimization techniques, as shown in the following.

**Assertion** **3.** *The optimal*$t_{\mathrm{Tr}}^{*}$is computed by the expression below:

(30) $$t_{\mathrm{Tr}}^{*}=\frac{d_{\mathrm{App}}}{\frac{B}{\ln2}W_{0}\left[\left(-\frac{g_{s}^{2}f_{\mathrm{MD}}c_{\mathrm{App}}}{\left(N_{0}+I\right)BT_{\mathrm{Sav}}}-\frac{1}{e}\right)2^{-\frac{d_{\mathrm{App}}}{BT_{\mathrm{Sav}}}}\right]+1+\frac{d_{\mathrm{App}}}{T_{\mathrm{Sav}}}}.$$

*where*$W_{0}\left(\cdot \right)$*is the Lambert Wfunction* [35].

**Proof.**  First, recall that $T_{\mathrm{Sav}}$ in (12) is the difference between the time that the task is executed locally and remotely. By letting the first-order derivative of the objective function of (29) be zero, we have:

(31) $$\begin{array}{cc} \; & \frac{T_{\mathrm{Max}}-t_{\mathrm{Lo}}}{g_{s}^{2}}\left\{\left[\frac{1}{t_{\mathrm{Tr}}-T_{\mathrm{Sav}}}+\frac{t_{\mathrm{Tr}}}{{\left(t_{\mathrm{Tr}}-T_{\mathrm{Sav}}\right)}^{2}}\right]h\left(\frac{d_{\mathrm{App}}}{t_{\mathrm{Tr}}}\right)+\frac{t_{\mathrm{Tr}}}{t_{\mathrm{Tr}}-T_{\mathrm{Sav}}}h^{\prime }\left(\frac{d_{\mathrm{App}}}{t_{\mathrm{Tr}}}\right)\right\}\\ \; & \;\;\;\;\;\;\;\;\;\;\;\;\;\;+f_{\mathrm{MD}}c_{\mathrm{App}}\frac{T_{\mathrm{Max}}-t_{\mathrm{Lo}}}{{\left(t_{\mathrm{Tr}}-T_{\mathrm{Sav}}\right)}^{2}}=0. \end{array}$$

It can be simplified as:

(32) $$\begin{array}{c} T_{\mathrm{Sav}}h\left(\frac{d_{\mathrm{App}}}{t_{\mathrm{Tr}}}\right)-t_{\mathrm{Tr}}\left(t_{\mathrm{Tr}}-T_{\mathrm{Sav}}\right)h^{\prime }\left(\frac{d_{\mathrm{App}}}{t_{\mathrm{Tr}}}\right)-g_{s}^{2}f_{\mathrm{MD}}c_{\mathrm{App}}=0, \end{array}$$

(33) $$\begin{array}{c} t_{\mathrm{Tr}}h\left(\frac{d_{\mathrm{App}}}{t_{\mathrm{Tr}}}\right)-g_{s}^{2}f_{\mathrm{MD}}c_{\mathrm{App}}=\left(t_{\mathrm{Tr}}-T_{\mathrm{Sav}}\right)\left[h\left(\frac{d_{\mathrm{App}}}{t_{\mathrm{Tr}}}\right)+t_{\mathrm{Tr}}h^{\prime }\left(\frac{d_{\mathrm{App}}}{t_{\mathrm{Tr}}}\right)\right], \end{array}$$

(34) $$\begin{array}{cc} \; & \left(N_{0}+I\right)Bt_{\mathrm{Tr}}\left(2^{\frac{d_{\mathrm{App}}}{Bt_{\mathrm{Tr}}}}-1\right)-g_{s}^{2}f_{\mathrm{MD}}c_{\mathrm{App}}\\ \; & \;\;\;\;\;=\left(t_{\mathrm{Tr}}-T_{\mathrm{Sav}}\right)\left[\left(N_{0}+I\right)B\left(2^{\frac{d_{\mathrm{App}}}{Bt_{\mathrm{Tr}}}}-1\right)-\frac{\left(N_{0}+I\right)d_{\mathrm{App}}\ln2\;2^{\frac{d_{\mathrm{App}}}{Bt_{\mathrm{Tr}}}}}{t_{\mathrm{Tr}}}\right]. \end{array}$$

By denoting $u≜\frac{d_{\mathrm{App}}}{Bt_{\mathrm{Tr}}}$ and based on Equation (34), *u* satisfies:

(35) $$\begin{array}{cc} \; & \left(N_{0}+I\right)\left[d_{\mathrm{App}}\ln2\;2^{u}+BT_{\mathrm{Sav}}2^{u}-BT_{\mathrm{Sav}}\ln2\;u\;2^{u}\right]=g_{s}^{2}f_{\mathrm{MD}}c_{\mathrm{App}}+\left(N_{0}+I\right)BT_{\mathrm{Sav}}, \end{array}$$

(36) $$\begin{array}{cc} \; & 2^{u}\;\ln2\;\left(\frac{d_{\mathrm{App}}}{BT_{\mathrm{Sav}}}-\frac{1}{\ln2}-u\right)=\frac{g_{s}^{2}f_{\mathrm{MD}}c_{\mathrm{App}}}{\left(N_{0}+I\right)BT_{\mathrm{Sav}}}+1, \end{array}$$

(37) $$\begin{array}{cc} \; & 2^{\left(u-\frac{d_{\mathrm{App}}}{BT_{\mathrm{Sav}}}-\frac{1}{\ln2}\right)}\ln2\left(u-\frac{d_{\mathrm{App}}}{BT_{\mathrm{Sav}}}-\frac{1}{\ln2}\right)=\left(-\frac{g_{s}^{2}f_{\mathrm{MD}}c_{\mathrm{App}}}{\left(N_{0}+I\right)BT_{\mathrm{Sav}}e}-\frac{1}{e}\right)2^{-\frac{d_{\mathrm{App}}}{BT_{\mathrm{Sav}}}}, \end{array}$$

We can finally further derive that:

(38) $$\begin{array}{cc} \; & e^{\ln2\left(u-\frac{d_{\mathrm{App}}}{BT_{\mathrm{Sav}}}-\frac{1}{\ln2}\right)}\ln2\left(u-\frac{d_{\mathrm{App}}}{BT_{\mathrm{Sav}}}-\frac{1}{\ln2}\right)=\left(-\frac{g_{s}^{2}f_{\mathrm{MD}}c_{\mathrm{App}}}{\left(N_{0}+I\right)BT_{\mathrm{Sav}}e}-\frac{1}{e}\right)2^{-\frac{d_{\mathrm{App}}}{BT_{\mathrm{Sav}}}}. \end{array}$$

However, recall that the condition $g_{s}>g_{\mathrm{Th}}$ has already been discussed in Section 3.2, resulting in full offloading.

For $g_{s}\leq g_{\mathrm{Th}}$, by referring to the definition of the Lambert W function [35], *u* in Equation (38) is obtained as follows:

$$\begin{array}{cc} u= & \frac{1}{\ln2}\left\{W_{0}\left[\left(-\frac{g_{s}^{2}f_{\mathrm{MD}}c_{\mathrm{App}}}{\left(N_{0}+I\right)BT_{\mathrm{Sav}}e}-\frac{1}{e}\right)2^{-\frac{d_{\mathrm{App}}}{BT_{\mathrm{Sav}}}}\right]+1\right\}+\frac{d_{\mathrm{App}}}{BT_{\mathrm{Sav}}}, \end{array}$$

Therefore, the optimal time $t_{\mathrm{Tr}}^{*}$ readily follows. □

The optimal transmitting power $p_{\mathrm{Tr}}^{*}$ can be derived from Equation (4) accordingly. Finally, $\alpha^{*}$ in (25) can be redescribed as:

(39) $$\alpha^{*}=\frac{t_{\mathrm{Lo}}-T_{\mathrm{Max}}}{T_{\mathrm{Sav}}-t_{\mathrm{Tr}}^{*}}.$$

Overall, the solution in (30) and (39) indicates that if the channel is not good enough, offloading the computing tasks to the remote MEC will have a high energy cost; thus, the partial offloading strategy is the preferred decision.

Combining the above subsections, the optimal solution of the primal problem (16) is finally presented as (40). For channel conditions better than the threshold $g_{\mathrm{Th}}$ defined in (17), full offloading is carried out; for channel $g_{s}<g_{\mathrm{Th}}$, partial offloading is performed, and the offloading ratio $\alpha^{*}$ and transmission time $t_{\mathrm{Tr}}^{*}$ are optimally chosen to minimize the total energy cost while satisfying the hard time constraint $T_{\mathrm{Max}}$. Finally, the transmission power $p_{\mathrm{Tr}}^{*}$ can be derived from $t_{\mathrm{Tr}}^{*}$ by (4).

(40) $$\begin{array}{cc} \; & \left\{\begin{array}{c} t_{\mathrm{Tr}}^{*}=T_{\mathrm{Max}}-\frac{c_{\mathrm{App}}}{h_{\mathrm{MEC}}},\;\;\;\;\;\;\;\alpha^{*}=1,\;\;\mathrm{if}\;g_{s}>g_{\mathrm{Th}}\\ t_{\mathrm{Tr}}^{*}=\frac{d_{\mathrm{App}}}{\frac{B}{\ln2}W_{0}\left[\left(-\frac{g_{s}^{2}f_{\mathrm{MD}}c_{\mathrm{App}}}{\left(N_{0}+I\right)BT_{\mathrm{Sav}}}-\frac{1}{e}\right)2^{-\frac{d_{\mathrm{App}}}{BT_{\mathrm{Sav}}}}\right]+1+\frac{d_{\mathrm{App}}}{T_{\mathrm{Sav}}}},\;\alpha^{*}=\frac{t_{\mathrm{Lo}}-T_{\mathrm{Max}}}{T_{\mathrm{Sav}}-t_{\mathrm{Tr}}^{*}},\;\mathrm{if}\;g_{s}\leq g_{\mathrm{Th}} \end{array}\right. \end{array}$$

### 3.4. Special Cases of Full Offloading and Non Offloading

Here, we proceed to analyze the special cases of the optimization problem, to determine under what conditions the binary decisions of full offloading or non offloading can be performed.

#### 3.4.1. Optimality Condition of Total Offloading

Here, we investigate the conditions under which the optimum is to process all the application bits at the MEC server, i.e., $\alpha^{*}=1$. The condition is twofold: (1) $\alpha^{*}=1$ should be feasible, and (2) $\frac{\partial e_{{\mathrm{Tot}}^{*}}}{\partial \alpha^{*}}\leq 0,\forall \;0\leq \alpha^{*}\leq 1$.

According to the completion time constraint, the first condition (1) holds when $T_{\mathrm{Max}}\geq t_{\mathrm{Off}}^{*}$, and $t_{\mathrm{Off}}^{*}=t_{\mathrm{Tr}}^{*}+t_{\mathrm{Ser}}$, i.e., the time required to transmit the task to the MEC server plus the time for remote processing should not be larger than the maximum latency constraint.

Finally, the sufficient condition (2) can be expanded as:

(41) $$\begin{array}{cc} \frac{\partial e_{{\mathrm{Tot}}^{*}}}{\partial \alpha^{*}} & =\frac{t_{\mathrm{Tr}}^{*}}{g_{s}^{2}}h\left(\frac{d_{\mathrm{App}}}{t_{\mathrm{Tr}}^{*}}\right)-f_{\mathrm{MD}}c_{\mathrm{App}}\leq 0\Rightarrow e_{\mathrm{Off}}^{*}\leq e_{\mathrm{Lo}}. \end{array}$$

This condition holds when $g_{s}\geq g_{\mathrm{Th}}$ and $t_{\mathrm{Tr}}^{*}\geq T_{\mathrm{Sav}}$, which is equivalent to the situation where it will take less energy to offload the application, as discussed in Section 3.2.

#### 3.4.2. Optimality of Non Offloading

Here, we provide the necessary conditions under which the optimal solution is to process all task bits locally at the MD, i.e., $\alpha^{*}=0$. These conditions are twofold: (1) $\alpha^{*}=0$ should be feasible, and (2) $\frac{\partial e_{\mathrm{Tot}}^{*}}{\partial \alpha^{*}}{|}_{\alpha^{*}=0}\geq 0$.

According to the completion time constraint, the first condition (1) holds only if $T_{\mathrm{Max}}\geq t_{\mathrm{Lo}},$ i.e., the time for executing all the application bits locally at the MD should not violate the latency constraint.

On the other hand, we see from Equation (19) that $e_{\mathrm{Tot}}^{*}\left(\alpha^{*}\right)$ is equal to $f_{\mathrm{MD}}c_{\mathrm{App}}$ at $\alpha^{*}=0$ and is continuous within a certain interval containing $\alpha^{*}=0$. These two characteristics allow us to state that $e_{\mathrm{Tot}}^{*}\left(\alpha^{*}\right)$ is constant (i.e., not dependent on $\alpha^{*}$) within a certain interval containing $\alpha^{*}=0$; therefore, the second condition (2) holds only when:

(42) $$\frac{\partial e_{\mathrm{Tot}}^{*}}{\partial \alpha^{*}}=\frac{t_{\mathrm{Tr}}^{*}}{g_{s}^{2}}h\left(\frac{d_{\mathrm{App}}}{t_{\mathrm{Tr}}^{*}}\right)-f_{\mathrm{MD}}c_{\mathrm{App}}\geq 0\Rightarrow e_{\mathrm{Off}}^{*}\geq e_{\mathrm{Lo}}.$$

This condition indicates that if offloading within a time constraint costs more energy than local computing, the MD should rely on its own resources. This condition holds in the situation where $g_{s}\leq g_{\mathrm{Th}}$, and the proof is left for Appendix B.

To summarize the results of the above subsections, the offloading policy decisions are listed in Table 1, under different channel conditions and latency constraints.

## 4. Analysis of the System Parameters

In this section, we investigate how the system parameters affect the optimization problem, including the time constraint, task complexity, and processing capacity at MD. This not only enables us to understand how the offloading strategy operates under different system settings, but also helps the system-level investigations of practical MEC systems in further research.

### 4.1. The Latency Constraint $T_{\mathrm{Max}}$ and Feasibility of $\alpha^{*}$

The completion time constraint of (16) is $t_{\mathrm{Off}}\alpha +t_{\mathrm{Lo}}\left(1-\alpha \right)\leq T_{\mathrm{Max}}$, which is related to the available time budget (and formulated in terms of maximum allowed latency), and can be rewritten as a set of two detailed constraints as follows:

(43a) $$t_{\mathrm{Off}}\alpha \leq T_{\mathrm{Max}}\Rightarrow \alpha \leq \frac{T_{\mathrm{Max}}}{t_{\mathrm{Off}}},$$

(43b) $$t_{\mathrm{Lo}}\left(1-\alpha \right)\leq T_{\mathrm{Max}}\Rightarrow \alpha \geq 1-\frac{T_{\mathrm{Max}}}{t_{\mathrm{Lo}}}.$$

Therefore, the minimum and maximum values of variable α can be defined as follows:

(44a) $$\alpha \geq \alpha_{\mathrm{Min}},\alpha_{\mathrm{Min}}=\max\left\{0,1-\frac{T_{\mathrm{Max}}}{t_{\mathrm{Lo}}}\right\},$$

(44b) $$\alpha \leq \alpha_{\mathrm{Max}},\alpha_{\mathrm{Max}}=\min\left\{1,\frac{T_{\mathrm{Max}}}{t_{\mathrm{Off}}}\right\}.$$

The primal problem (16) is feasible only if $\alpha_{\mathrm{Max}}\geq \alpha_{\mathrm{Min}}$. As shown in Equation (44a) and Equation (44b), $\alpha_{\mathrm{Max}}$ and $\alpha_{\mathrm{Min}}$ can be viewed as linear functions of $T_{\mathrm{Max}}$, and they are upper and lower bounded by one and zero, respectively. Next, we investigate how $T_{\mathrm{Max}}$ impacts the offloading policy in different channel conditions.

#### 4.1.1. Channel Gain $g_{s}>g_{\mathrm{Th}}$

**Figure 2:**
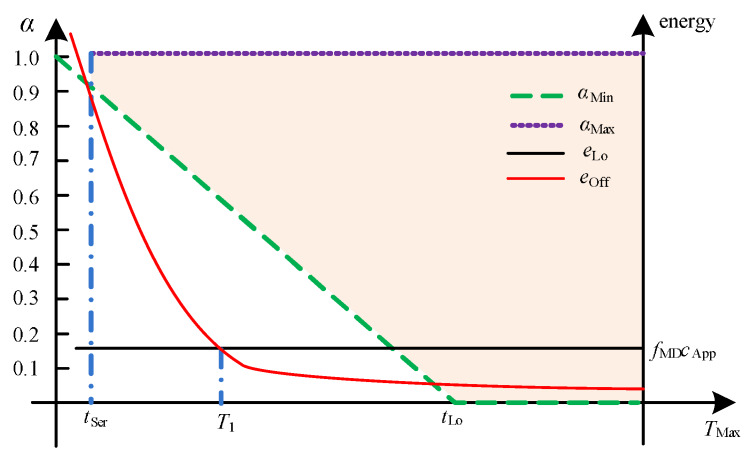
Dependence of $\alpha_{\mathrm{Max}}$, $\alpha_{\mathrm{Min}}$, and $e_{\mathrm{Tot}}^{*}$ versus $T_{\mathrm{Max}}$ for $g_{s}>g_{\mathrm{Th}}$.

In Figure 2, it can be clearly seen that it obtains the lowest value of $T_{\mathrm{Max}}$, under which a full offloading strategy will save energy. Let us denote such a lowest value by $T_{1}$. According to the closed form expressions of $t_{\mathrm{Tr}}^{*},e_{\mathrm{Off}}$ and $e_{\mathrm{Lo}}$, $T_{1}$ can be calculated as follows:

(45) $$\begin{array}{cc} \; & \left\{\begin{array}{c} t_{\mathrm{Tr}}^{*}=T_{\mathrm{Max}}-t_{\mathrm{Ser}}\\ \frac{t_{\mathrm{Tr}}^{*}}{g_{s}^{2}}h\left(\frac{d_{\mathrm{App}}}{t_{\mathrm{Tr}}^{*}}\right)=f_{\mathrm{MD}}c_{\mathrm{App}} \end{array}\right.\Rightarrow T_{\mathrm{Max}}=T_{1}=2^{\frac{d_{\mathrm{App}}}{B}}-\frac{g_{s}^{2}f_{\mathrm{MD}}c_{\mathrm{App}}}{(N_{0}+I)B}+t_{\mathrm{Ser}}. \end{array}$$

As seen in Figure 2, in the situation where $t_{\mathrm{Ser}}\leq T_{\mathrm{Max}}\leq T_{1}$, the full offloading strategy has to be carried out to meet the tight latency constraint at the expense of high energy consumption. If $T_{1}\leq T_{\mathrm{Max}}\leq t_{\mathrm{Lo}}$, the full offloading can save both time and energy. If $t_{\mathrm{Lo}}\leq T_{\mathrm{Max}}$, offloading will take advantage of the relaxed time constraint, extending the transmission time to save more energy.

#### 4.1.2. Channel Gain $g_{s}<g_{\mathrm{Th}}$

**Figure 3:**
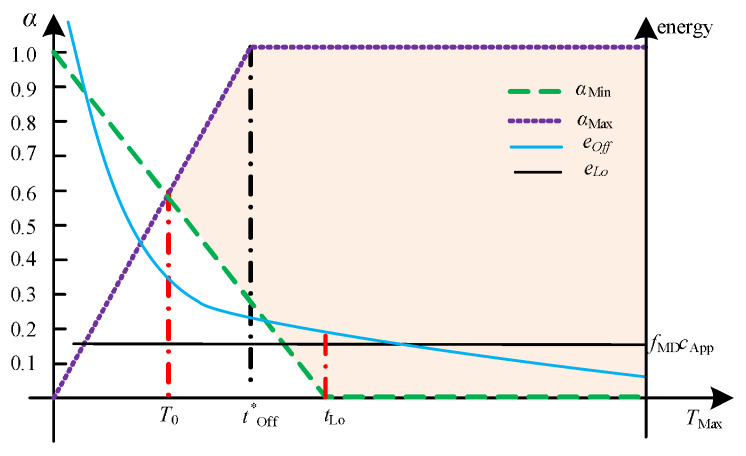
Dependence of $\alpha_{\mathrm{Max}}$, $\alpha_{\mathrm{Min}}$, and $e_{\mathrm{Tot}}^{*}$ versus $T_{\mathrm{Max}}$ for $g_{s}\leq g_{\mathrm{Th}}$.

In Figure 3, it can be observed that, for any $t_{\mathrm{Off}}$ and $t_{\mathrm{Lo}}$, there exists the lowest value of $T_{\mathrm{Max}}$ (also called the minimum admissible latency) under which $\alpha^{*}$ in Equation (16) becomes unfeasible. Let us denote the lowest value by $T_{0}$. By referring to (44a) and (44b), $T_{0}$ has to satisfy that:

(46) $$1-\frac{T_{0}}{t_{\mathrm{Lo}}}=\frac{T_{0}}{t_{\mathrm{Off}}}\Rightarrow T_{0}=\frac{t_{\mathrm{Off}}t_{\mathrm{Lo}}}{t_{\mathrm{Off}}+t_{\mathrm{Lo}}}.$$

In fact, as shown in Figure 3, $T_{0}$ is the *x*-coordinate of the intersection-point of the two constraint functions $\alpha_{\mathrm{Max}}\left(T_{\mathrm{Max}}\right)$ and $\alpha_{\mathrm{Min}}\left(T_{\mathrm{Max}}\right)$. Furthermore, it can be readily verified from (46) that:

- $T_{0}\leq t_{\mathrm{Lo}}$, $T_{0}\leq t_{\mathrm{Off}}$;
- $T_{0}>T_{\mathrm{Ser}}$.

where $t_{\mathrm{Lo}}$ is the time that is needed to do all the processing locally at the MD and $t_{\mathrm{Ser}}$ is the time that would be needed to do all the processing remotely at the MEC server, as defined in (5) and (9).

For the policy specified in the previous section,

(47) $$t_{\mathrm{Off}}^{*}=t_{\mathrm{Tr}}^{*}+t_{\mathrm{Ser}},$$

where $t_{\mathrm{Tr}}^{*}$ is defined in (30). Now, $T_{0}^{*}$ can be evaluated from (46):

(48) $$T_{0}^{*}=\frac{t_{\mathrm{Off}}^{*}t_{\mathrm{Lo}}}{t_{\mathrm{Off}}^{*}+t_{\mathrm{Lo}}},$$

which is the minimum admissible latency under this policy. For the time constraint $T_{\mathrm{Max}}$ satisfying $T_{0}^{*}\leq T_{\mathrm{Max}}\leq t_{\mathrm{Lo}}$, the partial offloading is optimal to minimize the total energy consumption for meeting the latency constraint at the same time. If $t_{\mathrm{Lo}}\leq T_{\mathrm{Max}}$, the local computing strategy is optimal in terms of energy and time consumption. Note that in the situation where $t_{\mathrm{Ser}}\leq T_{\mathrm{Max}}\leq T_{0}^{*}$, the full offloading is optimal and can meet the tight latency constraint at the expense of high energy consumption.

Interestingly, when the time budget (i.e., the maximum allowed latency $T_{\mathrm{Max}}$) is equal to the minimum affordable latency $T_{0}$, partial offloading is required, and the distribution of the task size is given by:

(49) $$\alpha_{\mathrm{Min}}=\alpha_{\mathrm{Max}}\Rightarrow \frac{t_{\mathrm{Lo}}}{t_{\mathrm{Off}}^{*}+t_{\mathrm{Lo}}}.$$

where the previous expressions were obtained by finding the point where functions $\alpha_{\mathrm{Max}}\left(T_{\mathrm{Max}}\right)$ and $\alpha_{\mathrm{Min}}\left(T_{\mathrm{Max}}\right)$ intersect.

### 4.2. The Channel Gain Threshold as a Function of the System Parameters

For those computation-intensive applications, it is inefficient to run it locally, because too much energy is consumed per CPU cycle at the MD or because the application spends too many CPU cycles per bit. In this situation, the full offloading strategy is more energy and time efficient for MDs. Here, we introduce the parameter bit-wise application complexity, which is defined as $C_{\mathrm{App}}≜\frac{c_{\mathrm{App}}}{d_{\mathrm{App}}}$, and the channel gain threshold $g_{\mathrm{Th}}$ can be expressed as a function of $f_{\mathrm{MD}}$ and $C_{\mathrm{App}}$ as follows:

(50) $$g_{\mathrm{Th}}=\sqrt{\frac{(N_{0}+I)B}{f_{\mathrm{MD}}}\left(\frac{1}{h_{\mathrm{MD}}}-\frac{1}{h_{\mathrm{MEC}}}\right)\left[2^{\frac{1}{BC_{\mathrm{App}}\left(\frac{1}{h_{\mathrm{MD}}}-\frac{1}{h_{\mathrm{MEC}}}\right)}}-1\right]}.$$

**Figure 4:**
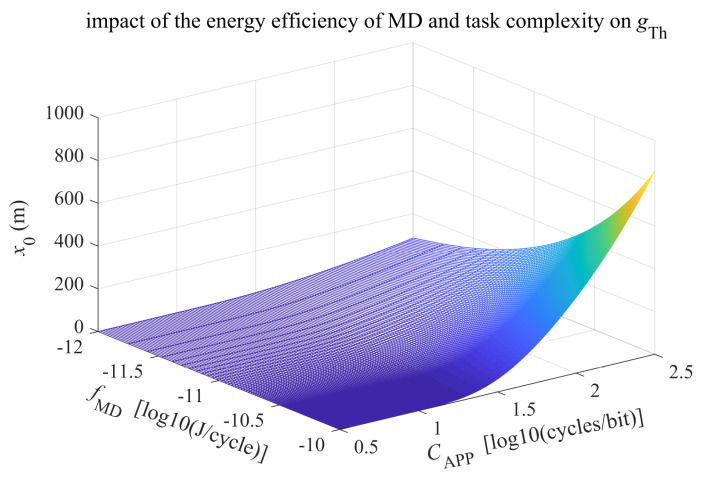
The effective coverage of MEC versus the energy efficiency of MD and task complexity ($h_{\mathrm{MD}}=1$ GHz, $h_{\mathrm{MEC}}=10$ GHz, B=5 MHz, and $N_{0}+I=-145$ dBm).

Figure 4 shows the radius of the effective coverage $x_{0}$ of an MEC server installed in a picocell base station for full offloading, based on the relationship between path loss [36] and channel gain $L_{T}=-20\log g_{\mathrm{Th}}$. As shown in Figure 4, with the energy efficiency of MD becoming weaker and the application job becoming increasingly complicated, the effective coverage region decreases; thus, more MDs will benefit from MEC in the cell.

Moreover, for asymptotically large $C_{\mathrm{App}}$, the channel gain threshold $g_{\mathrm{Th}}$ in (50) can be further approximated as follows:

(51) $$g_{\mathrm{Th}}\approx \sqrt{\frac{\left(N_{0}+I\right)}{f_{\mathrm{MD}}C_{\mathrm{App}}\;\ln2}}.$$

**Figure 5:**
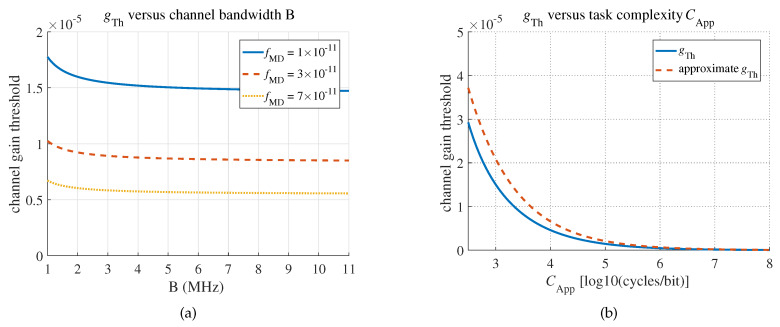
Channel gain threshold $g_{\mathrm{Th}}$. (**a**) $g_{\mathrm{Th}}$ versus B. (**b**) $g_{\mathrm{Th}}$ versus $C_{\mathrm{App}}$.

As shown in Figure 5, $g_{\mathrm{Th}}$ is largely determined by the local computing power and task complexity.

In conclusion, the proposed method is applicable to the emerging computational-intensive and/or delay-sensitive applications, i.e., novel AI applications from sectors such as the Industrial Internet of Things, intelligent robots, smart cities, and smart homes.

## 5. Simulation Results

The following provides simulation results to illustrate the performance of the proposed offloading strategy. We first consider the scenario where the wireless picocell base station had a coverage radius of 500 m and the base station antenna was located inside the building. According to the path loss model for the residential environment, the path loss model is expressed as:

(52) $$L_{T}=20\log f_{c}\left[\mathrm{MHz}\right]+28\log x_{d}\left[m\right]+L_{f}\left(n_{f}\right)-28.$$

where $f_{c}=2$ GHz is the carrier frequency; $x_{d}$ is the distance between the mobile user and the wireless base station; $L_{f}\left(n_{f}\right)=4n_{f}$ (dB) is the floor penetration factor for the ITU-Rmodel (13.2) [36], and the number of floors $n_{f}=1$. We considered the computational task to be generated by a face recognition application, and the task profile refers to [37,38]. The computation profile refers to [38,39]. Moreover, as the mobile devices are evolving to become smarter and the resolution of the images and videos is getting higher, we set the data size for the task to $d_{\mathrm{App}}=5000$ KB. The simulation parameters are summarized in Table 2. These parameters were used as the default in the subsequent simulations unless explicitly specified. Beforehand, we calculated $t_{\mathrm{Ser}}=1$ s, $t_{\mathrm{Lo}}=10$ s, $T_{\mathrm{Sav}}=9$ s, $e_{\mathrm{Lo}}=0.1$ J.

The channel gain threshold was calculated from Table 2 where $g_{\mathrm{Th}}=4.0\times 10^{-5}$, according to Equation (17). The relationship between channel gain and path loss was $L_{T}=-20\log g_{\mathrm{Th}}$; thus, the MEC’s full offloading coverage radius $x_{0}=200$ m, which is denoted as the effective coverage in the rest of the paper.

In addition, we also list the numerical values of MEC’s full offloading coverage radius $x_{0}$ for situations of different channel interference strengths and bit-wise complexity $C_{\mathrm{App}}$, as shown in Table 3. For an MEC system, developers can use these values to choose the right location of the MEC servers so as to make full use of their computing resources.

**Figure 6:**
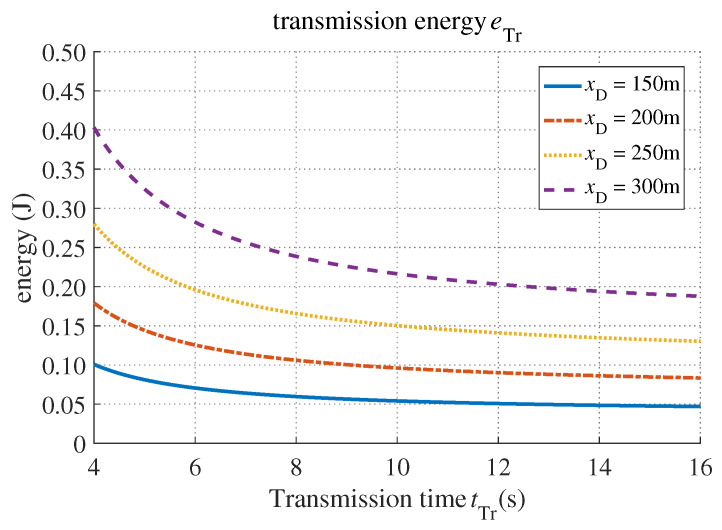
The transmission energy versus transmission time.

Figure 6 shows the relationship between transmission time and transmission energy consumed by the MD. Note that the energy spent was $\frac{N_{0}Bt_{\mathrm{Tr}}}{g_{s}^{2}}\left(2^{\frac{d_{\mathrm{App}}}{Bt_{\mathrm{Tr}}}}-1\right)$. As the transmission time increased, it required less energy for the MD to transmit task data to the MEC server. In addition, when the MD got closer to the BS, the channel condition became better, i.e., the channel gain improved, and it cost the MD less energy to offload the computing task.

**Figure 7:**
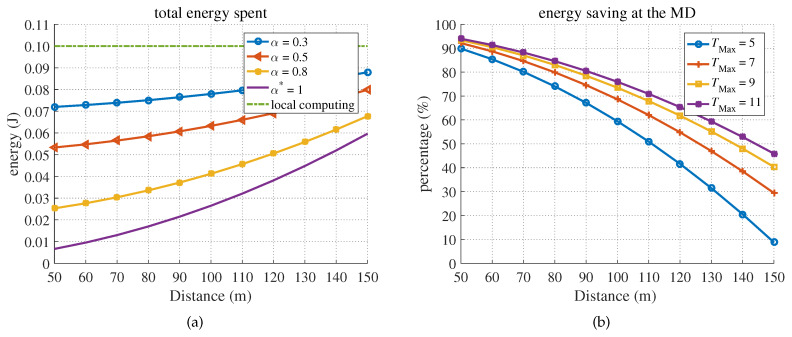
The energy consumption and percentage of energy savings due to offloading under different latency constraints versus the distance between the BS and mobile device (MD). (**a**) Energy consumption. (**b**) Energy savings.

Next, for $g_{s}>g_{\mathrm{Th}}$, we verified that the full offloading policy was optimal in terms of minimizing energy consumption, as shown in Figure 7.

Figure 7a shows the energy consumption for different distances between the BS and MD in the MEC-effective coverage of $g_{s}>g_{\mathrm{Th}}$. As shown in the figure, for $g_{s}>g_{\mathrm{Th}}$, the full offloading policy was optimal for saving energy, which corresponded to the optimum solution of (16). In such a situation and $T_{\mathrm{Max}}\geq T_{1}$, full offloading energy was always lower than local computing energy. Moreover, only the task data size, the task complexity, and the CPU computing capability of MDs could affect the energy consumption and latency of local computing. Therefore, the changes in the distance between the MD and BS or the task completion time requirement did not influence the performance of local computing. Figure 7b shows the energy savings as a percentage for the case of full offloading. Note that in the case of full offloading, the offloading time is $T_{\mathrm{Max}}-t_{\mathrm{Ser}}$. As shown in the figure, if the latency deadline became less hard, and the distance from the BS decreased (the channel gain increased), the percentage of energy savings would increase, as expected.

In the situation in which the MD was out of the MEC-effective coverage for which $g_{s}\leq g_{\mathrm{Th}}$, partial offloading was preferred, and we continued to evaluate performance of the energy-optimal policy under various settings.

**Figure 8:**
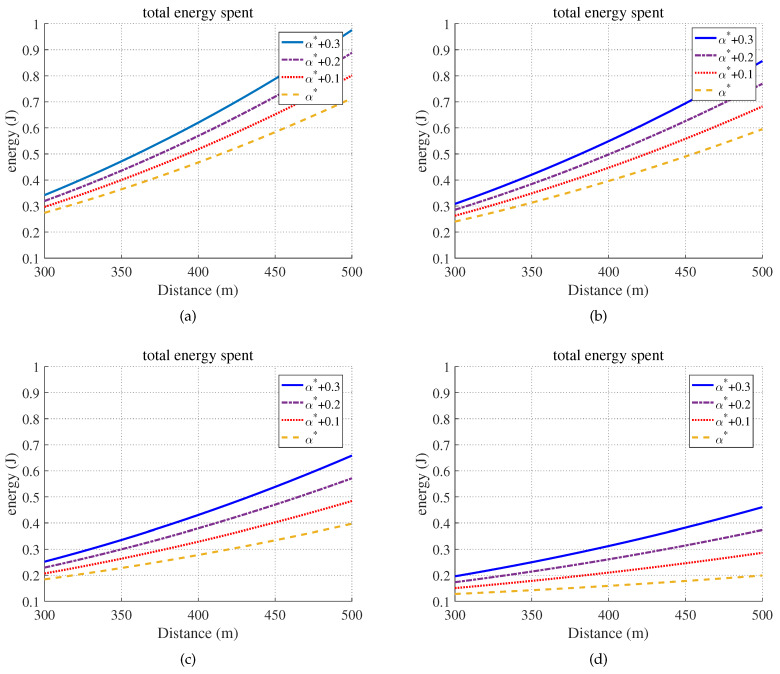
The completion energy consumption versus the distance from the BS under different latency constraints. (**a**) $T_{\mathrm{Max}}=6.5$ s. (**b**) $T_{\mathrm{Max}}=7.5$ s. (**c**) $T_{\mathrm{Max}}=8.5$ s. (**d**) $T_{\mathrm{Max}}=9.5$ s.

Figure 8 demonstrates that the proposed partial offloading strategy was optimal for minimizing the energy consumption under the hard delay constraint, and the offloading fraction $\alpha^{*}=\frac{t_{\mathrm{Lo}}-T_{\mathrm{Max}}}{T_{\mathrm{Sav}}-t_{\mathrm{Tr}}^{*}}$ increased as the latency deadline became more stringent. As shown in the figure, the total energy consumption increased as the distance from the BS increased.

**Figure 9:**
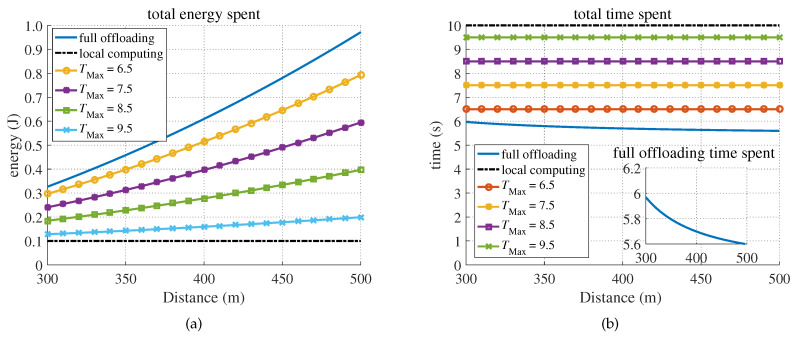
The completion energy and time consumption versus the distance from the BS under different latency constraints. (**a**) Energy consumption. (**b**) Time consumption.

Figure 9 also shows the energy and time consumption for different distances between the BS and MD for the case of partial offloading. For comparison, full offloading and local computing are included in Figure 9. When $g_{s}<g_{\mathrm{Th}}$, full offloading consumed more energy than partial offloading and local computing under the same delay constraint, as shown in Section 3. Moreover, the offloading energy increased as the distance from the BS increased, as shown in Figure 9a. We can observe from Figure 9b that the full offloading time was less than the partial offloading and local computing time. In fact, to meet the time constraint of $T_{\mathrm{Max}}\leq t_{\mathrm{Lo}}$, it was necessary for the MD to distribute a part of the computing load to the MEC server; thus, the partial offloading strategy always cost more energy than local computing, as shown in Figure 9a. Additionally, to minimize the energy consumption at the MD, the proportion of the offloaded application decreased with increasing distance. Therefore, more time was consumed on local computing. To compensate for the additional time loss of local computing, the offloading data rate $r_{\mathrm{Tr}}$ must be increased, yielding a shorter full offloading time $t_{\mathrm{Off}}^{*}$, as shown in Figure 9b.

**Figure 10:**
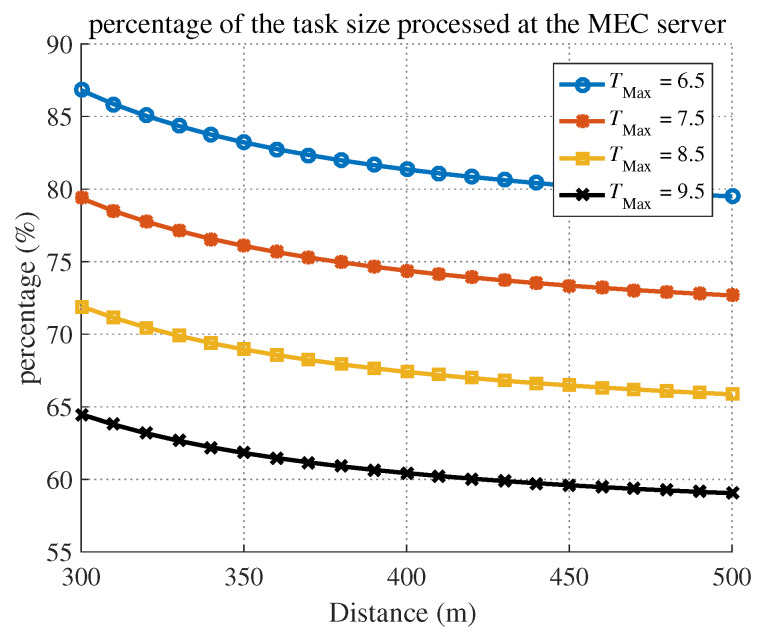
Percentage of the task remotely processed at the MEC server versus the distance from the BS under different latency constraints.

Figure 10 shows the percentage of a task to be remotely processed at the MEC server versus the distance from the BS. The task offloading percentage decreased as the distance from the BS increased, as expected. Note that as the latency constraint became tighter, the offloading percentage increased, and the percentage was also affected by the distance from the BS, which coincided with the optimal solution in (39).

## 6. Conclusions

This paper presented an offloading strategy for optimizing communication and computation resources usage in an MEC scenario. An energy consumption minimization problem was formulated with the latency constraint. A channel gain threshold of binary offloading and partial offloading was derived, through analyzing the optimization problem. Since the problem was non-convex and difficult to solve directly, we decomposed the original non-convex problem into two sub-problems to obtain the optimal solution in closed form expression. The analytical solution also yielded new understandings of the inherent trade-off between the energy consumption and latency.

For future work, we will extend the strategy to the multi-MD offloading scenario in a multi-access wireless environment. In addition, we are interested in considering a more dynamic model in which the MD may join and leave the MEC server’s coverage within a foreseeable period, in which the mobility patterns might play an important role in the problem formulation.

## Figures and Tables

**Table 1:** Offloading policy decisions.

	Latency	$t_{\mathrm{Max}}<t_{\mathrm{Lo}}$	$t_{\mathrm{Max}}\geq t_{\mathrm{Lo}}$
Channel			
$g_{s}>g_{\mathrm{Th}}$		Full offloading	Full offloading
$g_{s}\leq g_{\mathrm{Th}}$		Partial offloading	Non offloading

**Table 2:** Parameter setup.

Parameter	Value
B	5 MHz
$N_{0}$	−174 dBm
*I*	−145 dBm
$d_{\mathrm{App}}$	5000 kBytes
$c_{\mathrm{App}}$	1000 Megacycles
$f_{\mathrm{MD}}$	$1\times 10^{-11}$ J/cycle
$h_{\mathrm{MD}}$	1 GHz
$h_{\mathrm{MEC}}$	10 GHz

**Table 3:** Radius of effective coverage $x_{0}$.

${x_{0}}^{*}$	${C_{\mathrm{App}}}_{**}$	200	700	1300	2000	4000	6000
*I*							
−145 dBm		200	433	606	759	1085	1333
−135 dBm		62	134	187	235	335	412
−125 dBm		20	42	59	74	106	130
−115 dBm		6	13	19	23	33	41
−105 dBm		2	4	6	7	10	13

* Unit of *x*_0_:m. ** Unit of *C*_App_:cycles/bit.

## Appendix A. Monotonicity of *e*_Tot_ as a Function of *t*_Tr_

The total energy consumption of the MD includes both local computing consumption and partial offloading consumption, which is expressed as:

(A1) $$\begin{array}{cc} e_{\mathrm{Tot}} & =p_{\mathrm{Tr}}t_{\mathrm{Tr}}\alpha +f_{\mathrm{MD}}c_{\mathrm{App}}\left(1-\alpha \right) \end{array}$$

(A2) $$\begin{array}{cc} \; & =\frac{t_{\mathrm{Tr}}}{g_{s}^{2}}h\left(\frac{d_{\mathrm{App}}}{t_{\mathrm{Tr}}}\right)\alpha +f_{\mathrm{MD}}\;c_{\mathrm{App}}\left(1-\alpha \right) \end{array}$$

To prove that $e_{\mathrm{Tot}}$ is a monotonically decreasing function of $t_{\mathrm{Tr}}$, we need to compute the partial derivative in which:

(A3) $$\begin{array}{cc} \frac{\partial e_{\mathrm{Tot}}}{\partial \;t_{\mathrm{Tr}}} & =\alpha \left[\frac{(N_{0}+I)B}{g_{s}^{2}}\left(2^{\frac{d_{\mathrm{App}}}{Bt_{\mathrm{Tr}}}}-1\right)-\frac{\left(N_{0}+I\right)d_{\mathrm{App}}\ln2}{g_{s}^{2}t_{\mathrm{Tr}}}2^{\frac{d_{\mathrm{App}}}{Bt_{\mathrm{Tr}}}}\right] \end{array}$$

(A4) $$\begin{array}{cc} \; & =\frac{\alpha (N_{0}+I)B}{g_{s}^{2}}\left(2^{\frac{d_{\mathrm{App}}}{Bt_{\mathrm{Tr}}}}-\frac{d_{\mathrm{App}}\ln2}{Bt_{\mathrm{Tr}}}2^{\frac{d_{\mathrm{App}}}{Bt_{\mathrm{Tr}}}}-1\right). \end{array}$$

For notational simplicity, let $v≜\frac{d_{\mathrm{App}}}{Bt_{\mathrm{Tr}}}$, and let $f\left(v\right)=2^{v}-v\;2^{v}\ln2-1$, then:

(A5) $$\frac{\partial e_{\mathrm{Tot}}}{\partial (t_{\mathrm{Tr}})}=\frac{\alpha (N_{0}+I)B}{g_{s}^{2}}\left(2^{v}-v\ln2\;2^{v}-1\right).$$

It can be noticed that $f\left(v\right){|}_{v=0}\;=\;0$, besides:

(A6) $$f^{\prime }\left(v\right)=-{\left(\ln2\right)}^{2}\;v\;2^{v}\leq 0,\;v\geq 0.$$

Thus, f(v)≤0 for v≥0. Finally, by noticing that:

(A7) $$\frac{\partial \;e_{\mathrm{Tot}}}{\partial \;t_{\mathrm{Tr}}}=\frac{\alpha (N_{0}+I)B}{g_{s}^{2}}f\left(v\right)\leq 0,$$

the lemma is proved.

## Appendix B. The Existence of Channel Threshold *g*_Th_

Let us consider the case in which the offloading task can be finished in the same time interval as the local computing time in which $t_{\mathrm{Max}}=t_{\mathrm{Lo}}$. By referring to Equations (10) and (12), one finds that $t_{\mathrm{Tr}}=T_{\mathrm{Sav}}$. Therefore, the energy consumption in the transmitting stage can be written as a function of channel gain $g_{s}$ as follows:

(A8) $$\begin{array}{cc} e_{0}\left(g_{s}\right) & =\frac{1}{g_{s}^{2}}h\left(\frac{d_{\mathrm{App}}}{t_{\mathrm{Tr}}}\right)\\ \; & =\frac{1}{g_{s}^{2}}\left(N_{o}+I\right)BT_{\mathrm{Sav}}\left(2^{\frac{d_{\mathrm{App}}}{BT_{\mathrm{Sav}}}}-1\right). \end{array}$$

Next, one may compare $e_{0}\left(g_{s}\right)$ with local computing energy consumption, which is $f_{\mathrm{MD}}c_{\mathrm{App}}$. As $e_{0}$ is an inverse proportional function of $g_{s}$ in (A8), there exists a threshold $g_{\mathrm{Th}}$, such that for $g_{s}>g_{\mathrm{Th}}$, the offloading energy is less than the local $f_{\mathrm{MD}}c_{\mathrm{App}}$. Therefore,

(A9) $$g_{\mathrm{Th}}=\sqrt{\frac{\left(N_{0}+I\right)BT_{\mathrm{Sav}}\left(2^{\frac{d_{\mathrm{App}}}{BT_{\mathrm{Sav}}}}-1\right)}{f_{\mathrm{MD}}c_{\mathrm{App}}}}.$$

For channel conditions better than threshold $g_{\mathrm{Th}}$, full offloading with $\alpha^{*}=1$ is optimal because in this case, transmitting the data to the MEC server always consumes less energy than local computing under the same latency constraint.

The result above can now be extended to prove that full offloading ($\alpha^{*}=1$) is optimal for the general channel condition $g_{s}>g_{\mathrm{Th}}$. The constraints of $T_{\mathrm{Max}}<t_{\mathrm{Lo}}$ and $T_{\mathrm{Max}}>t_{\mathrm{Lo}}$ are discussed as follows.

1. If $T_{\mathrm{Max}}<t_{\mathrm{Lo}}$, then offloading has to be performed because the time constraint cannot be totally satisfied by full local computing. Now, assume if there exists some fraction yet to be locally processed, then it must not be the optimal choice because offloading requires less energy than local computing for $g_{s}>g_{\mathrm{Th}}$ under the same time constraint.
2. If $T_{\mathrm{Max}}>t_{\mathrm{Lo}}$, then $t_{\mathrm{Tr}}>T_{\mathrm{Sav}}$. By Lemma 1, offloading will further reduce energy consumption due to the longer transmission time, which is advantageous to local computing.

## Appendix C. Proof the Monotonicity of *e*_Tot_ with Respect to *α* When *g*_s_ < *g*_Th_

Based on (30), it is obvious that $\frac{\partial t_{\mathrm{Tr}}^{*}}{\partial (g_{s}^{2})}\geq 0$ and $t_{\mathrm{Tr}}^{*}$ is equal to $T_{\mathrm{Sav}}$ at the channel condition threshold $g_{s}=g_{\mathrm{Th}}$; thus, if $g_{s}\leq g_{\mathrm{Th}}$, we have $t_{\mathrm{Tr}}^{*}\leq T_{\mathrm{Sav}}$. In addition, $e_{\mathrm{Tot}}^{*}$ satisfies that $\frac{\partial e_{\mathrm{Tot}}^{*}}{\partial t_{\mathrm{Tr}}^{*}}\leq 0$, which was proven in Section 3.3. Therefore, if $t_{\mathrm{Tr}}^{*}\leq T_{\mathrm{Sav}}$, we can calculate that:

(A10) $$\begin{array}{cc} \frac{\partial e_{\mathrm{Tot}}}{\partial \alpha } & =\frac{\left(N_{0}+I\right)Bt_{\mathrm{Tr}}}{g_{s}^{2}}\left(2^{\frac{d_{\mathrm{App}}}{Bt_{\mathrm{Tr}}}}-1\right)-f_{\mathrm{MD}}c_{\mathrm{App}} \end{array}$$

(A11) $$\begin{array}{cc} \; & \geq \frac{\left(N_{0}+I\right)BT_{\mathrm{Sav}}}{g_{s}^{2}}\left(2^{\frac{d_{\mathrm{App}}}{BT_{\mathrm{Sav}}}}-1\right)-f_{\mathrm{MD}}c_{\mathrm{App}} \end{array}$$

(A12) $$\begin{array}{cc} \; & \geq \frac{\left(N_{0}+I\right)BT_{\mathrm{Sav}}}{g_{\mathrm{Th}}^{2}}\left(2^{\frac{d_{\mathrm{App}}}{BT_{\mathrm{Sav}}}}-1\right)-f_{\mathrm{MD}}c_{\mathrm{App}}\\ \; & =0 \end{array}$$
